# Laboratory performance of antimicrobial susceptibility testing for GLASS pathogens among participants in the EQuAfrica programme, 2021‒2025

**DOI:** 10.1371/journal.pgph.0007478

**Published:** 2026-10-27

**Authors:** Olga Perovic, Beatrice van der Puije, Jane Y. Carter, Felix Humwa, Rubeina Badat, Dennis Mwiti, Marshagne Smith, Fatime Poulo Ly, Cheikh Fall, Yakhya Dièye, Patience Dabula, Rene S. Hendriksen, Yenew Kebede Tebeje, Pascale Ondoa

**Affiliations:** 1 National Institute for Communicable Diseases (NICD) a division of National Health Laboratory Service, Johannesburg South Africa; 2 School of Pathology, University of Witwatersrand, Johannesburg, South Africa; 3 African Society for Laboratory Medicine (ASLM), Addis Ababa, Ethiopia; 4 African Field Epidemiology Network (AFENET), Kampala, Uganda; 5 Amref Health Africa (Amref), Nairobi, Kenya; 6 Pôle de Microbiologie, Institut Pasteur de Dakar, Dakar, Sénégal; 7 National Food Institute (DTU Food), at Technical University of Denmark, Lyngby, Denmark; 8 Africa Centre for Diseases Control and Prevention (Africa CDC), Addis Ababa, Ethiopia; 9 The Global Fund to Fight AIDS, Tuberculosis and Malaria, Geneva, Switzerland; 10 Amsterdam Institute for Global Health and Development, Department of Global Health, University of Amsterdam, Amsterdam, the Netherlands; Aga Khan University, PAKISTAN

## Abstract

The External Quality Assessment (EQA) for Africa (EQuAFRICA) project aimed at building regional capacity to provide a One Health bacteriology proficiency testing (PT) programme for reference and sentinel laboratories supporting antimicrobial resistance (AMR) surveillance and submission of data to the World Health Organization Global Antimicrobial Resistance and Use Surveillance System (WHO GLASS). We analysed the performance of participating laboratories in pathogen specific identification (ID) and qualitative antimicrobial susceptibility testing. The programme covered all GLASS and other bacterial pathogens of importance. One survey was distributed in 2021 and 2023 and two surveys in 2022, 2024 and 2025, with five isolates per survey. Participants were selected among reference and designated sentinel AMR laboratories from the 14 Fleming Fund recipient countries in Africa. The Clinical Microbiology Proficiency Testing (CMPT) marking scheme was used to score performance. The number of invited, enrolled and responding laboratories differed by survey; an average of 85% of enrolled laboratories submitted results. Participants scored 83% for organism ID across all cycles. Microbiology laboratories from the human health sector represented the majority in all cycles (75%). The average scores for antibiotic susceptibility testing (AST) was 73%, no participants that scored zero. Critical AST errors with clinical implications for directed treatment included vancomycin and cefoxitin for staphylococci. Our manuscript highlights that the use of EQA programmes to detecting gaps in laboratory processes and maintaining the quality and accuracy of AST is vital for effective AMR surveillance and control. This report illustrates that poorly performed AST at the beginning of the programme improved over the time. Building sustainable capacity for bacteriology testing, including AST, is critically important at all laboratory levels across Africa, from routine clinical and surveillance sites to national AMR reference laboratories.

## Background

Antimicrobial resistance (AMR) surveillance is critical for monitoring AMR trends, providing notifications for multidrug resistant pathogens and supporting infection prevention and control measures. Accurate AMR data inform treatment decisions, guide national and local policies and direct the development of new antimicrobial drugs. Most laboratories in Africa rely on conventional culture-based phenotypic diagnostics for bacterial identification (ID) and antimicrobial susceptibility testing (AST) based on disk diffusion of broth dilution), which requires standardised and robust quality assured procedures. However, laboratories in low- and middle-income countries (LMICs) including those at reference levels, often lack the capacity and resources to meet requirements for basic bacteriology testing procedures [1]. The World Health Organization (WHO) has established the Global Antimicrobial Resistance and Use Surveillance System (GLASS) programme, to provide a standardised approach to the collection, analysis, interpretation and sharing of AMR data, articulated around eight priority bacterial pathogens [2,3].

External quality assessment (EQA), based on proficiency testing (PT) panels, is a critical component of quality assured laboratory testing [4–6] but has remained challenging to implement for AMR detection. Good quality AMR PT panels are complex to design and to produce, as they need to comprehensively cover priority GLASS pathogens and include challenges for pathogen isolation, identification, isolation and antibiotic susceptibility testing [6]. The World Health Organization Regional Office for Africa (WHO AFRO) AMR PT programme was established in 2018 to address the limited access to AMR EQA material in the region [7]; however, the coverage of this subsidized initiative was limited to 23 countries and failed to demonstrate a lasting impact on the performance of participating facilities. Overall, the performance of the few laboratories participating in available EQA programmes remains sub-optimal [7], in a context where continuous quality improvement activities, quality management system and ISO 15189 accreditation remain under- resourced and poorly implemented [8]. The lack of competence to conduct AMR detection and surveillance, further compounds the narrow capability of African laboratories to test and report on GLASS priority pathogens [9,10], with negative clinical and public health implications.

The External Quality Assessment (EQA) for Africa (EQuAFRICA) project was launched in 14 countries in the context of a Fleming Fund UK Aid Fleming Fund (FF) regional grant for Africa to build an effective and sustainable regional One Health AMR proficiency testing (PT). EQuAfrica was designed as a coalition of technical partners including the National Institute for Communicable Diseases (NICD), the Technical University of Denmark (DTU), Amref Health Africa (Amref) -Kenya and the Institute Pasteur de Dakar (IPD), Senegal managed by the African Society for Laboratory Medicine (ASLM) and under the strategic leadership of the Africa Centres for Disease Control and Prevention (ACDC). To address the weaknesses of previous AMR PT programmes, EQuAfrica included capacity building and quality management implementation components at the attention of participating laboratories. The overall performance of participating laboratories and the effectiveness of the EQuAfrica programme are critical to the continuous improvement of the regional AMR PT. In addition to this programmatic evaluation, the disaggregated analysis of the various techniques involved in quality AST have the potential to bring insights into the specific technical challenges faced by the laboratories participating to EQA and to contribute to more targeted capacity building.

The aim of this study was to analyse the qualitative performance of the laboratories participating to EQuAFRICA laboratories between 2021 and 2025, across pathogen isolation, identification, AST and identification of mechanisms of resistance. Our finding intended to inform interventions for building laboratory system capacity for AMR surveillance.

## Materials and methods

### Ethics statement

As no human or animal material was used for this programme and the PT programme followed ISO 17043 standard, no ethical permission was required. Based on ISO 17043 standard requirement, individual participants data remained confidential and protected.

#### EQuAFRICA programme design.

The PT programme was designed and overseen by a technical advisory committee (TAC) comprising experts in microbiology and public health in animal, human and environmental science, who provided guidance on technical, administrative and scientific components of the programme. Besides the WHO GLASS priority pathogens, the TAC decided other organisms of importance for the region should be included. Enrolment of participants was directed by ASLM as the grant recipient. ASLM managed the informatics and data integration for the EQA programme through developed digital platform. All challenges were submitted to five to ten referee laboratories in South Africa, Kenya and Senegal to obtain a consensus on results.

#### PT panel design.

Each PT panel was made up of five different mainly GLASS pathogens, with a distinct composition in each cycle. The GLASS pathogens are *Acinetobacter* spp., *E. coli; K. pneumoniae; P. aeruginosa; S. aureus; S. pneumoniae; N. meningitidis; H. influenzae; Salmonella spp.; Salmonella enterica serovar* Typhi*; Salmonella enterica serovar* Paratyphi A*; Shigella spp.; N. gonorrhoeae* was not used in the surveys [3]. The different case scenarios were developed for the same isolates when applied to the different sectors, human, animal, and environment. Antibiotic panels for testing were selected based on Clinical and Laboratory Standards Institute (CLSI) and/or European Committee on Antimicrobial Susceptibility Testing (EUCAST) [11–14] guidelines for the first-line treatment options for each organism in the survey; in each survey over the five year period, and per survey. Each survey required evaluation of AST for at least two organisms. Reporting of resistance phenotypes: extended spectrum of beta-lactamases (ESBLs), Carbapenem resistant Enterobacterales CREs, methicillin resistant Staphylococcus aureus MRSA and vancomycin resistant enterococci VRE were evaluated where appropriate. Participants were provided with relevant National Collection of Type Cultures (NCTC) culture strains for the purpose of running quality tests. The overall successful performance threshold was set at 80% for acceptable responses and results within each category ID and AST.

To manage programme operations, an informatics system was developed by ASLM and deployed to capture results, collate data, generate reports, and facilitate cycle and shipment communications.

#### Participating laboratories and registration into the PT programme.

Participants were selected among reference and designated sentinel AMR laboratories from the 14 Fleming Fund recipient countries in Africa. Participating laboratories were nominated by national authorities prioritising AMR reference laboratories and major surveillance sites in the human, animal and environmental health sectors to embrace a One Health approach.

Demographic and capacity information about the laboratories (such as tier level, affiliation, equipment or accreditation, supplementary material) were collected through a standardised registration form at first participation.

#### Scoring and laboratory performance.

We implemented the Clinical Microbiology Proficiency Testing (CMPT) marking scheme from the PT programme at the Department of Pathology and Laboratory Medicine, University of British Columbia, Vancouver, Canada [15]. All graded areas required ≥80% referee consensus before participating laboratories’ results were evaluated. Assessment criteria: acceptable scores – score of 4 or 3; unacceptable scores – score of 1 or 0; not evaluated (NE) – assigned if a valid reason for not submitting a response for a specific graded area was provided. Overall acceptable target: ≥ 80% participants reporting correct responses per graded area. Scoring was done for each component of the challenge as follows in Table 1.

**Table 1:** Evaluation criteria and allocated scores per challenge.

Score	Gram stain response
**4**	Reporting correct Gram stain reaction and correct morphology, e.g., correctly reporting an expected result of Gram-negative bacilli
**3**	No criteria for evaluation
**2**	Separator - Never assigned
**1**	Reporting correct Gram stain reaction and incorrect morphology, e.g., reporting Gram-negative cocci for expected response of Gram-negative bacilli
**0**	Reporting incorrect Gram stain reaction with correct or incorrect morphology, e.g., reporting Gram-positive bacilli for expected response of Gram-negative bacilli
	No response without providing a valid reason (e.g., lack of reagents or expired reagents) for not reporting
**NE**	A valid reason for not responding is provided
**Score**	**Final identification response**
**4**	Correct Genus and species result reported, e.g., correctly reporting an expected result of *Klebsiella pneumoniae*
**3**	Correct Genus, no species specified, e.g., reporting *Klebsiella* species for an expected response of *Klebsiella pneumoniae*
**2**	Separator - Never assigned
**1**	Reporting correct Genus with incorrect species reported, e.g., reporting *Klebsiella oxytoca* for an expected response of *Klebsiella pneumoniae*
	Reporting an unnamed or unspecified microorganism, e.g., reporting Gram-negative bacilli or Gram-negative fermenter for an expected response of *Klebsiella pneumoniae*
**0**	Incorrect identification, e.g., reporting *Acinetobacter baumannii* for an expected response of *Klebsiella pneumoniae*
	No growth - possible indication of incorrect culture methods and or conditions
	No response without providing a valid (e.g., lack of reagent or expired reagents) reason for not reporting
**NE**	A valid reason for not responding is provided
**Score**	**Response for individual requested antibiotic**
**4**	Reporting correct expected response
**3**	An expected response of “Intermediate” reported as “Susceptible” (mE)
	An expected response of “Intermediate” reported as “Resistant” (mE)
	An expected response of “Susceptible” reported as “Intermediate” (mE)
	An expected response of “Resistant” reported as “Intermediate” (mE)
**2**	Separator - Never assigned
**1**	An expected response of “Susceptible” reported as “Resistant” (ME)
	Not reporting a vital antibiotic that is requested, e.g., not reporting oxacillin/cefoxitin for *Staphylococcus aureus* (ME)
	Reporting antibiotic results where the guideline used is not stated (ME)
	Reporting antibiotic results where a final organism identification is not provided/known (ME)
	Reporting an antibiotic result where there is no guideline (CLSI/EUCAST etc.) for interpretation available, e.g., reporting a vancomycin disk susceptibility result for *Staphylococcus aureus* (there are no disk interpretation guidelines available). (ME)
**0**	An expected response of “Resistant” reported as “Susceptible” (VME)
	If “Final identification” score = 0, AST is not evaluated; automatically scored “0”

mE: Minor error, ME: Major error and VME: Very major error.

Each requested antibiotic is individually scored using the evaluation criteria above. The average is then calculated and a score out of 4 is assigned for the “AST result” graded area.

The scoring of AST was based on qualitative criteria for susceptible, intermediate and resistant organisms using either CLSI or EUCAST guidelines:

- Correct (scored 4/4)–the participant’s results agreed with those from the referee laboratories.
- Minor error (scored 3/4)–the participant results indicated an ‘intermediate’ susceptibility result, and the referee laboratories reported either a susceptible or a resistant result.
- Major error (scored 1/4)–the participant results indicated the microorganism as resistant to the antimicrobial drug, whereas the referee laboratories reported the microorganism as susceptible. In this category antibiotic testing methods and reporting errors were assigned as major.
- Very major error (scored 0/4)–the most serious error; the participant results indicated the microorganism was susceptible to the antimicrobial drug, where the referee laboratories indicated that the microorganism was resistant.

Acceptable laboratory performance is presented as the proportion (%) of participants that achieved scores of 3 or 4 (acceptable performance) for pathogen ID and AST.

#### Results report and capacity building.

Results are reported as in two tiers: one for each laboratory and another a summary of aggregated results of all participants. Post-cycle online workshops were organized and hosted by the regional EQA providers after each cycle to provide technical assistance for all participating laboratories. Strengths and challenges were addressed and provision of technical advice to address challenges was provided. As the part of the overall programme, mentors (subject matter experts) were also allocated by ASLM to provide on-site country support to laboratories and sentinel surveillance sites.

#### Data analysis.

Descriptive statistics were calculated for pathogen, facility characteristics, and performance by analyte and cycle and presented as frequencies and percentages. For antibiotic performance evaluation, chi-square tests were used to assess differences in acceptable performance across cycles for each antibiotic. For antibiotics where expected cell counts were less than 5, Fisher’s exact test was employed. All tests were two-sided, and a p-value <0.05 was considered statistically significant. To identify factors associated with acceptable performance for ID and AST outcomes, we performed stepwise logistic regression. The unit of analysis was at the laboratory-cycle-pathogen level, where performance scores were aggregated by taking the mean numeric score per laboratory, cycle and pathogen combination. Acceptable performance was defined as a mean score ≥3, while non-acceptable performance was defined as a mean score of ≤3. Univariate logistic regression was first conducted for all candidate predictors, including facility characteristics (network tier, self-reported capability, sector, participation cycles), AST guideline used, automation method, and pathogen characteristics (Gram stain, genus, WHO risk classification, GLASS pathogen status, clinical case type, and specimen types). Variables with a univariate p-value <0.1 were entered into the multivariate logistic regression model. Odds ratios (OR) and 95% confidence intervals (CI) were calculated for all predictors in both univariate and multivariate models. The multivariate model was built using stepwise selection with entry and exit criteria of p < 0.1. All analyses were performed using R software version 4.2.3 (R Core Team, 2022)

## Results

The programme was implemented in two phases: 2021–2022 and 2023–2025. Distributions of surveys and pathogens are shown in Fig 1. There was one survey in 2021, two surveys in 2022, one survey in 2023 and two surveys in 2024 and 2025, respectively Fig 1.

**Fig 1:**
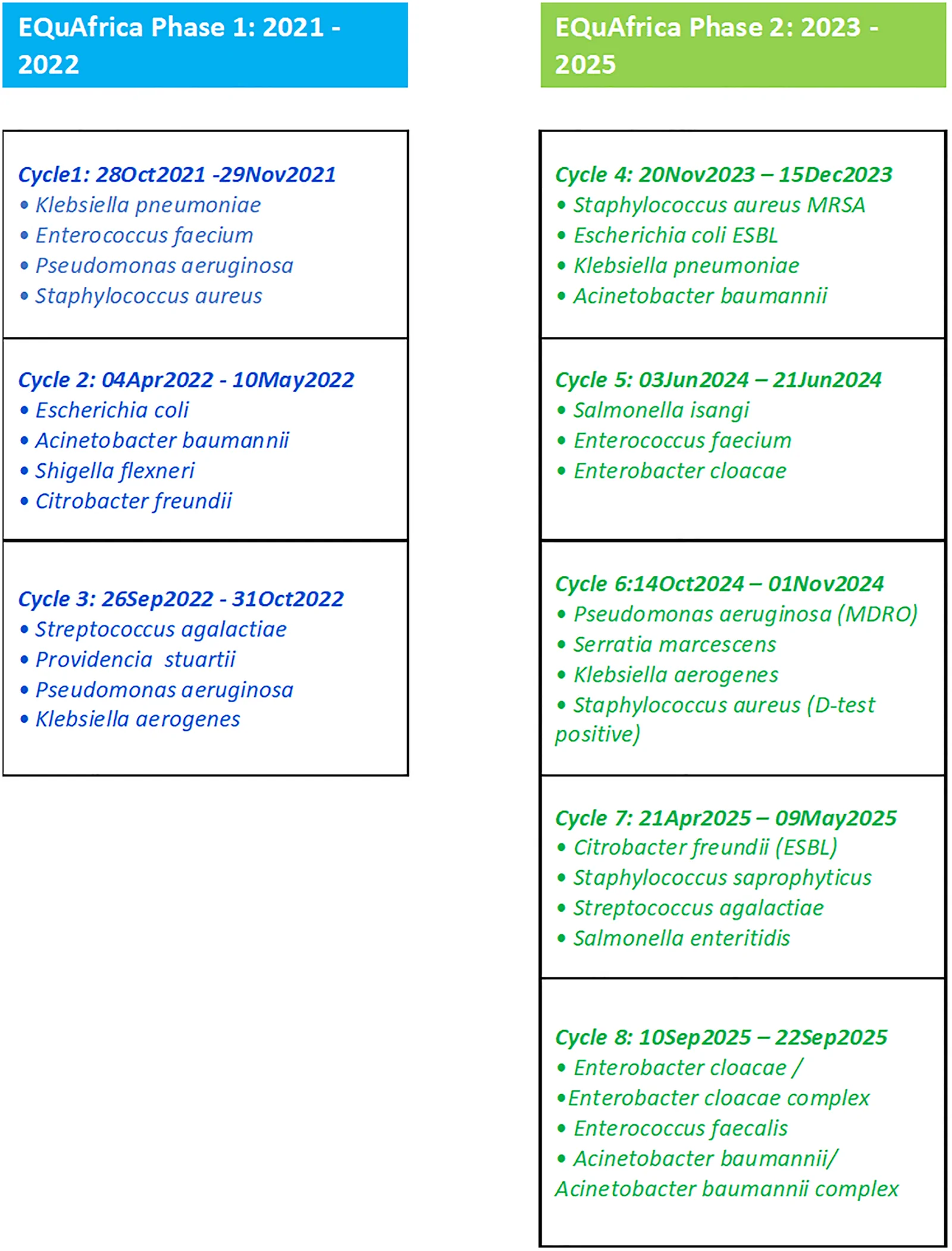
Pathogen distribution per cycle.

A total of 32 pathogen isolates were evaluated across the eight EQA cycles (Table 2 and S1 Table). All challenges were embedded into clinical case scenarios. Gram-negative organisms comprised 69% (n = 22) of the isolates, while Gram-positive organisms comprised 31% (n = 10). The most frequently represented genus included *Klebsiella* (13%, n = 4), *Staphylococcus* (13%, n = 4), *Pseudomonas* (9.4%, n = 3), and *Acinetobacter* (9.4%, n = 3). According to the WHO risk classification, 31% (n = 10) were classified as critical priority pathogens, 25% (n = 8) as high priority, and 9.4% (n = 3) as medium priority. The majority (78%, n = 25) were GLASS-listed pathogens.

**Table 2:** Pathogens characteristics and clinical case scenarios.

Characteristic	N = 32^1^
**Gram**	
Gram Negative	22 (69%)
Gram Positive	10 (31%)
**Genus**	
*Acinetobacter*	3 (9.4%)
*Citrobacter*	2 (6.3%)
*Enterobacter*	2 (6.3%)
*Enterococcus*	3 (9.4%)
*Escherichia*	2 (6.3%)
*Klebsiella*	4 (13%)
*Listeria*	1 (3.1%)
*Providencia*	1 (3.1%)
*Pseudomonas*	3 (9.4%)
*Salmonella*	2 (6.3%)
*Serratia*	1 (3.1%)
*Shigella*	2 (6.3%)
*Staphylococcus*	4 (13%)
*Streptococcus*	2 (6.3%)
**WHO risk**	
Critical	10 (31%)
High	8 (25%)
Medium	3 (9.4%)
NA	11 (34%)
**GLASS pathogen**	
GLASS	25 (78%)
Non-GLASS	7 (22%)
**Clinical case scenarios**	
Community associated infections	9 (31%)
Healthcare associated infections	18 (62%)
Recurrent community infections	2 (6.9%)
**Human specimen**	
CSF, blood, sterile aspirates	5 (25%)
Sputum/tracheal	5 (25%)
Stool	3 (15%)
Urine	7 (35%)
**Animal specimen**	
Milk	4 (22%)
Tissue	10 (56%)
Urine/Caecal	4 (22%)
**Environmental specimen**	
Food	3 (19%)
Swabs	6 (38%)
Waste water	7 (44%)

^1^n (%).

### Participating laboratories

A total of 340 participant laboratories were included in the EQuAfrica analysis. Table 3 shows the numbers and characteristics of the participating laboratories. Most laboratories were sentinel Laboratories (54%, n = 184), while 46% (n = 156) were national public health and reference laboratories (Table 2). Regarding self-reported bacteriology and AST capability, 62% (n = 189) were classified as basic, 30% (n = 91) as intermediate, and 7.6% (n = 23) as advanced. Most laboratories were from the human health sector (76%, n = 258), followed by the animal health (21%, n = 73), environmental health (1.5%, n = 5), and food safety (1.2%, n = 4) sectors. The distribution of participation across EQuAfrica cycles showed that 55% (n = 187) of laboratories participated in five or more cycles, while 20% (n = 67) participated in two cycles, 14% (n = 49) in three cycles, 9.1% (n = 31) in one cycle, and 1.8% (n = 6) in four cycles. Regarding AST guidelines, 61% (n = 206) of laboratories reported using CLSI, 11% (n = 36) used EUCAST, 5.9% (n = 20) used CA-SFM, while 23% (n = 77) varied their AST guidelines across cycles, and 0.3% (n = 1) did not specify. For pathogen identification methods, 84% (n = 284) of laboratories varied their use of automated methods across cycles, 11% (n = 39) consistently used non-automated methods, 4.7% (n = 16) consistently used automated methods, and 0.3% (n = 1) did not specify.

**Table 3:** Laboratory characteristics.

Characteristic	N = 340^1^
**Surveillance Function**	
National Public Health and Reference Laboratory	156 (46%)
Sentinel Laboratory	184 (54%)
**Self-reported bacteriology testing and AST capability**	
Advanced	23 (7.6%)
Basic	189 (62%)
Intermediate	91 (30%)
**Sector**	
Animal Health Laboratory	73 (21%)
Environmental Health Laboratory	5 (1.5%)
Food Safety Laboratory	4 (1.2%)
Human Health Laboratory	258 (76%)
**Number of laboratories participating in EQuAfrica cycles**	
1 Cycle	31 (9.1%)
2 Cycles	67 (20%)
3 Cycles	49 (14%)
4 Cycles	6 (1.8%)
5 or more cycles	187 (55%)
**Reported standard used for AST**	
CA-SFM	20 (5.9%)
CLSI	206 (61%)
EUCAST	36 (11%)
Not specified	1 (0.3%)
Varying	77 (23%)
**Utilisation of automated methods for pathogen identification or AST in any cycle**	
Automated	16 (4.7%)
Not automated	39 (11%)
Not specified	1 (0.3%)
Varying	284 (84%)

^1^n (%).

The average number of invited 201, enrolled 184 and responding 173 laboratories were presented for analysis. Increasing numbers of laboratories were invited to enrol in the program in the years 2024 and 2025, as shown in Table 3. On average, 158 laboratories with advanced capacity for ID and AST phenotypic and molecular testing, intermediate capacity with mainly phenotypic methods, and basic capacity with ID and manual disk methods participated in the programme.

### Overall performance by analyte and cycle

Table 4 presents the performance across AST, final organism identification (ID), and mechanisms of resistance by cycle. For final organism identification, acceptable performance showed a similar pattern, ranging from 58.0% (Cycle 3) to 85.3% (Cycle 4). Non-acceptable ID results were highest in Cycle 5 (28.4%) and Cycle 7 (27.2%), while not-evaluated results peaked in Cycle 3 (17.6%) and Cycle 5 (7.8%).

**Table 4:** Laboratories performance for IDs and AST stratified by cycles.

Final Identification				AST			Mechanism of Resistance		
cycle	Not acceptable	Not evaluated	Acceptable	Not acceptable	Not evaluated	Acceptable	Not acceptable	Not evaluated	Acceptable
Cycle 1	132 (22.6%)	15 (2.6%)	438 (74.9%)	117 (25%)	20 (4.3%)	331 (70.7%)	–	–	–
Cycle 2	147 (23.3%)	48 (7.6%)	435 (69%)	144 (28.6%)	11 (2.2%)	349 (69.2%)	–	–	–
Cycle 3	164 (24.4%)	118 (17.6%)	389 (58%)	118 (22%)	65 (12.1%)	353 (65.9%)	–	–	–
Cycle 4	90 (13.6%)	7 (1.1%)	563 (85.3%)	111 (21%)	15 (2.8%)	402 (76.1%)	–	–	–
Cycle 5	179 (28.4%)	49 (7.8%)	402 (63.8%)	136 (33.1%)	46 (11.2%)	229 (55.7%)	–	276 (100%)	–
Cycle 6	200 (21.6%)	34 (3.7%)	691 (74.7%)	223 (30.1%)	45 (6.1%)	473 (63.8%)	–	520 (100%)	–
Cycle 7	344 (27.2%)	43 (3.4%)	878 (69.4%)	323 (31.9%)	34 (3.4%)	657 (64.8%)	118 (11.7%)	450 (44.5%)	444 (43.9%)
Cycle 8	236 (20.4%)	64 (5.5%)	855 (74%)	234 (31.1%)	79 (10.5%)	440 (58.4%)	84 (11.2%)	563 (74.8%)	106 (14.1%)

For AST, acceptable performance ranged from 55.7% (Cycle 5) to 76.1% (Cycle 4), with notable improvement from Cycle 5 (55.7%) to Cycle 6 (63.8%) and Cycle 8 (58.4%). Non-acceptable AST results were highest in Cycle 5 (33.1%) and Cycle 7 (31.9%), while not-evaluated results peaked in Cycle 3 (12.1%) and Cycle 8 (10.5%).

Mechanisms of resistance were evaluated from Cycle 5 onward. In Cycle 5 and 6, all results (100%) were classified as acceptable. In Cycle 7, 43.9% were acceptable, 44.5% non-acceptable, and 11.7% not evaluated. In Cycle 8, acceptable performance for mechanisms decreased to 14.1%, while non-acceptable performance increased to 74.8%, with 11.2% not evaluated.

### Antibiotic performance across cycles

Performance varied substantially across antibiotics and cycles. Several antibiotics demonstrated consistently low performance across multiple cycles. Ciprofloxacin showed acceptable rates ranging from 38.8% (Cycle 5) to 85.5% (Cycle 3), with significant variation across cycles (p < 0.001). Gentamicin performance ranged from 0% (Cycle 5) to 73.3% (Cycle 2), with significant variation (p < 0.001). Trimethoprim-sulfamethoxazole performance ranged from 48.1% (Cycle 1) to 91.5% (Cycle 4), with significant variation (p < 0.001). Meropenem demonstrated relatively stable performance across cycles, ranging from 65.2% (Cycle 2) to 81.9% (Cycle 3), with significant variation (p < 0.001). Ciprofloxacin (p < 0.001), gentamicin (p < 0.001), and ceftriaxone (p < 0.001) all showed significant differences in acceptable rates across cycles. Antibiotics such as vancomycin, oxacillin, and cefoxitin were excluded from the analysis as per the study protocol.

The bar chart in the Fig 2 depicts the distribution of acceptable, unacceptable, and not-evaluated results for final organism identification (ID) across the eight EQA cycles. Acceptable ID performance varied considerably over time and by pathogen with Cycle 3 having the lowest performance in *Providencia stuartii.* No statistical significant differences in performance for pathogen ID were observed in Cycles 5 and 8.

**Fig 2:**
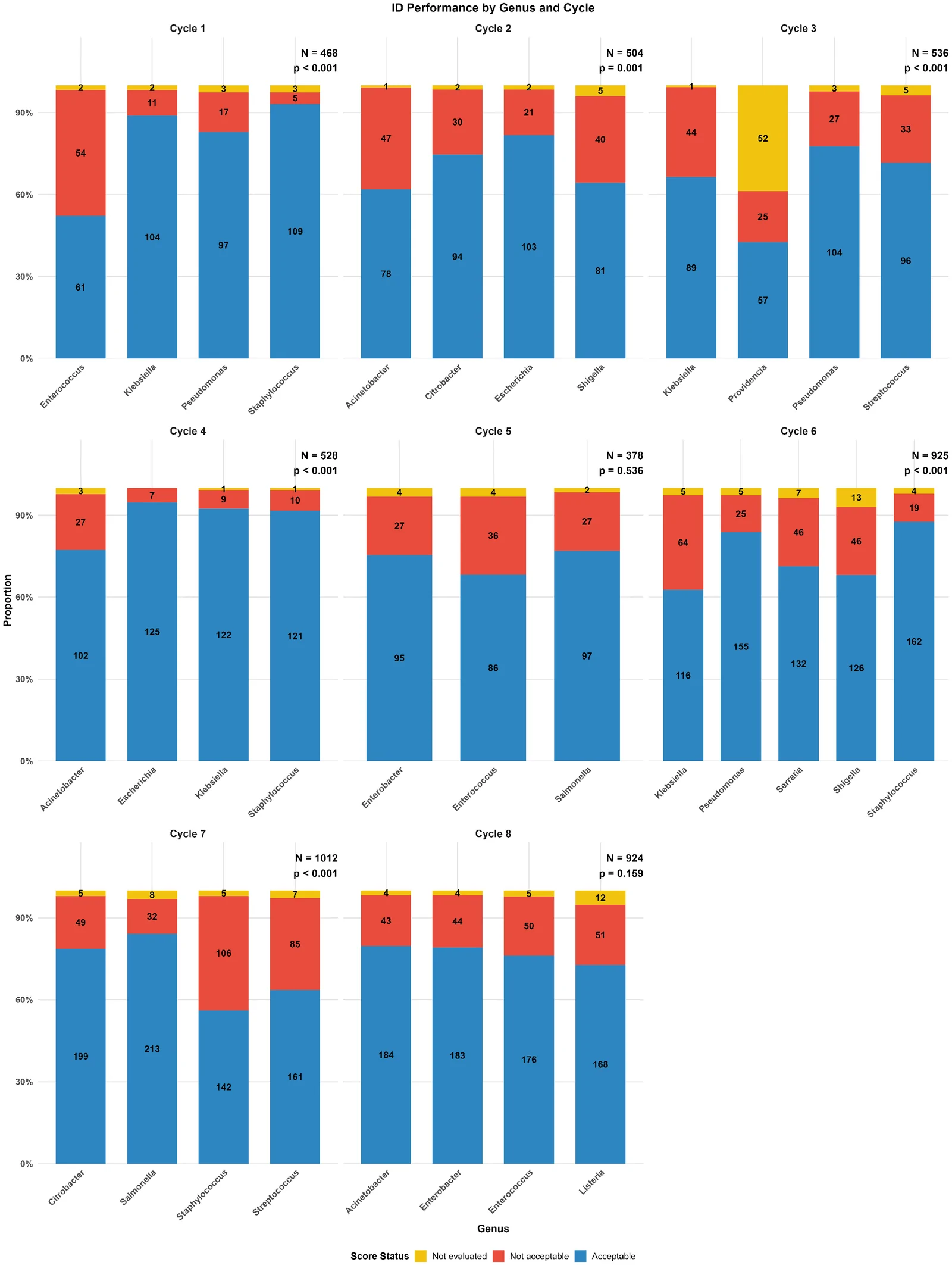
ID performance by cycle.

Fig 3, illustrates the performance in pathogen ID and antibiotic susceptibility testing (AST) across cycles. Acceptable AST rates by the pathogens varied significantly across the cycles apart from pathogens in Cycle 6 where there was no significant difference.

**Fig 3:**
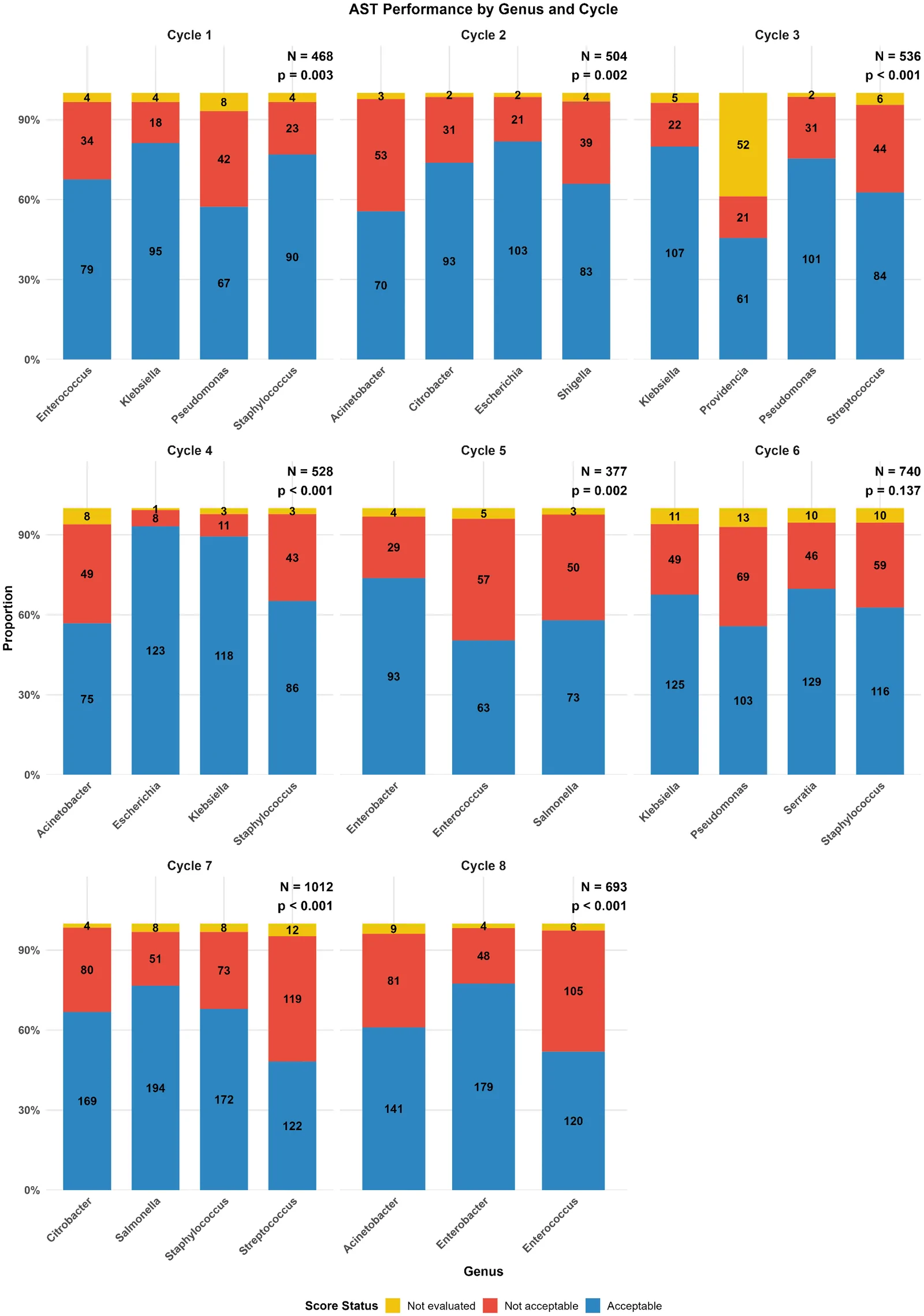
AST performance by pathogen and cycle.

The combined bar chart (Fig 4) shows the acceptable performance rates for ID and AST across the eight cycles. Both parameters exhibited similar overall patterns: performance improved from Cycle 1 to Cycle 4, dropped sharply in Cycle 5, and subsequently fluctuated at moderate levels. ID performance was consistently higher than AST in most cycles, with the largest gap observed in Cycle 4, 6 and 8. The most pronounced decline for both occurred in Cycle 5 (62.7% for AST and 69.2% for ID). The p-values for the trend (Cochran-Armitage test) were <0.001 for both parameters, confirming significant changes over time. These results highlight that performance was not uniform across cycles, with notable improvements in Cycle 4 and considerable drops in Cycle 5, possibly reflecting differences in pathogen complexity or laboratory familiarity with the tested organisms.

**Fig 4:**
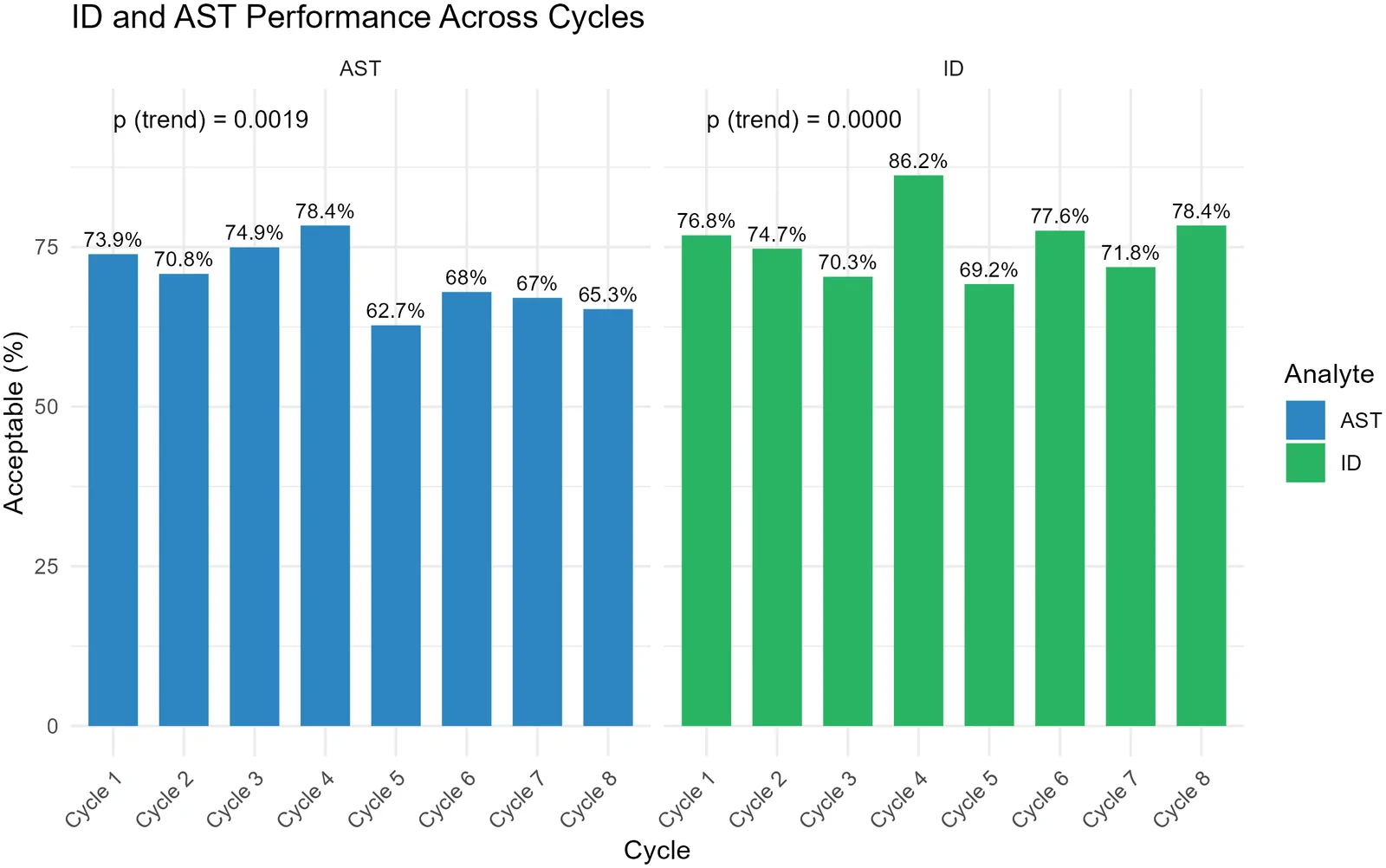
Combined ID and AST performance.

### Factors associated with acceptable performance

#### Final performance in pathogen ID.

Univariate and multivariate logistic regression analyses identified several factors significantly associated with acceptable final organism identification (ID) performance (Table 5). In multivariable analysis, sentinel laboratories were associated with lower odds of acceptable identification results compared to national public health and reference laboratories (adjusted OR [aOR]: 0.80, 95% CI: 0.65–0.99, p = 0.041). Laboratory self-reported bacteriology testing and AST capability was significantly associated with identification performance in univariate analysis (p < 0.001); however, after adjustment, neither basic (aOR: 1.26, 95% CI: 0.90–1.78, p = 0.2) nor intermediate (aOR: 1.33, 95% CI: 0.96–1.87, p = 0.092) capability levels remained significantly associated compared to advanced capability.

**Table 5:** Univariate and multivariate regression analysis.

	Final Identification Results									AST Results								
	Characteristics		Univariate				Multivariable			Characteristics		Univariate				Multivariable		
Variable	Acceptable, N = 6,038^1^	Not acceptable, N = 2,401^1^	N	OR^2^	95% CI^2^	p-value	OR^2^	95% CI^2^	p-value	Acceptable, N = 4,256^1^	Not acceptable, N = 2,196^1^	N	OR^2^	95% CI^2^	p-value	OR^2^	95% CI^2^	p-value
**Surveillance Function**			8,439			**0.005**						6,452			**<0.001**			
National Public Health and Reference Laboratory	3,543 (59%)	1,328 (55%)		*ref.*	—		*ref.*	—		2,591 (61%)	1,166 (53%)		*ref.*	—		*ref.*	—	
Sentinel Laboratory	2,495 (41%)	1,073 (45%)		1.15	1.04, 1.26		0.80	0.65, 0.99	**0.041**	1,665 (39%)	1,030 (47%)		1.37	1.24, 1.53		0.95	0.76, 1.18	0.6
**Self-reported bacteriology testing and AST capability**			7,190			**<0.001**						5,463			0.10			
Advanced	620 (12%)	175 (8.5%)		*ref.*	—		*ref.*	—		405 (11%)	195 (10%)		*ref.*	—		*ref.*	—	
Basic	2,352 (46%)	1,043 (51%)		1.57	1.31, 1.89		1.26	0.90, 1.78	0.2	1,653 (46%)	930 (49%)		1.17	0.97, 1.41		0.86	0.63, 1.19	0.4
Intermediate	2,159 (42%)	841 (41%)		1.38	1.15, 1.67		1.33	0.96, 1.87	0.092	1,516 (42%)	764 (40%)		1.05	0.87, 1.27		1.15	0.84, 1.56	0.4
**Sector**			8,439			**0.010**						6,452			**<0.001**			
Animal Health Laboratory	1,322 (22%)	568 (24%)		*ref.*	—		*ref.*	—		861 (20%)	585 (27%)		*ref.*	—		*ref.*	—	
Environmental Health Laboratory	46 (0.8%)	29 (1.2%)		1.47	0.90, 2.35		1.10	0.50, 2.32	0.8	32 (0.8%)	24 (1.1%)		1.10	0.64, 1.89		0.94	0.38, 2.29	0.9
Food Safety Laboratory	81 (1.3%)	19 (0.8%)		0.55	0.32, 0.89		0.51	0.20, 1.14	0.13	46 (1.1%)	28 (1.3%)		0.90	0.55, 1.44		0.90	0.41, 1.93	0.8
Human Health Laboratory	4,589 (76%)	1,785 (74%)		0.91	0.81, 1.01		0.58	0.46, 0.71	**<0.001**	3,317 (78%)	1,559 (71%)		0.69	0.61, 0.78		0.55	0.43, 0.69	**<0.001**
**Number of participations in EQuAfrica cycles**			8,439			**<0.001**						6,452			**<0.001**			
1 Cycle	82 (1.4%)	68 (2.8%)		*ref.*	—		*ref.*	—		50 (1.2%)	70 (3.2%)		*ref.*	—		*ref.*	—	
2 Cycles	396 (6.6%)	274 (11%)		0.83	0.58, 1.19		0.83	0.47, 1.46	0.5	260 (6.1%)	237 (11%)		0.65	0.43, 0.97		0.58	0.25, 1.28	0.2
3 Cycles	483 (8.0%)	252 (10%)		0.63	0.44, 0.90		0.52	0.29, 0.92	**0.025**	293 (6.9%)	255 (12%)		0.62	0.42, 0.93		0.44	0.19, 0.98	**0.047**
4 Cycles	66 (1.1%)	54 (2.2%)		0.99	0.61, 1.60		0.76	0.32, 1.80	0.5	45 (1.1%)	51 (2.3%)		0.81	0.47, 1.39		0.32	0.11, 0.92	**0.036**
5 or more Cycles	5,011 (83%)	1,753 (73%)		0.42	0.30, 0.59		0.22	0.13, 0.39	**<0.001**	3,608 (85%)	1,583 (72%)		0.31	0.22, 0.45		0.17	0.08, 0.38	**<0.001**
**Reported standard used for AST**			8,439			**<0.001**						6,452			**<0.001**			
CA-SFM	269 (4.5%)	116 (4.8%)		*ref.*	—		*ref.*	—		205 (4.8%)	104 (4.7%)		*ref.*	—		*ref.*	—	
CLSI	3,622 (60%)	1,356 (56%)		0.87	0.69, 1.09		0.84	0.55, 1.32	0.4	2,541 (60%)	1,237 (56%)		0.96	0.75, 1.23		1.00	0.61, 1.66	>0.9
EUCAST	472 (7.8%)	303 (13%)		1.49	1.15, 1.94		1.54	0.95, 2.55	0.084	331 (7.8%)	257 (12%)		1.53	1.15, 2.04		1.50	0.86, 2.67	0.2
Varying	1,675 (28%)	626 (26%)		0.87	0.69, 1.10		0.79	0.50, 1.27	0.3	1,179 (28%)	598 (27%)		1.00	0.78, 1.29		1.14	0.68, 1.93	0.6
**Utilization of automated methods for pathogen identification or AST in any cycle**			8,439			**<0.001**						6,452			**<0.001**			
Automated	215 (3.6%)	55 (2.3%)		*ref.*	—		*ref.*	—		156 (3.7%)	50 (2.3%)		*ref.*	—		*ref.*	—	
Not automated	252 (4.2%)	203 (8.5%)		3.15	2.23, 4.49		2.27	1.27, 4.19	**0.007**	158 (3.7%)	180 (8.2%)		3.55	2.44, 5.25		2.63	1.26, 5.75	**0.012**
Varying	5,571 (92%)	2,143 (89%)		1.50	1.12, 2.05		1.73	1.03, 3.04	**0.045**	3,942 (93%)	1,966 (90%)		1.56	1.14, 2.17		2.29	1.20, 4.64	**0.016**
**Gram**			6,810			**<0.001**						6,290			**<0.001**			
Gram Negative	3,493 (68%)	1,058 (63%)		*ref.*	—		*ref.*	—		3,042 (71%)	1,271 (62%)		*ref.*	—		*ref.*	—	
Gram Positive	1,640 (32%)	619 (37%)		1.25	1.11, 1.40		0.07	0.02, 0.22	**<0.001**	1,214 (29%)	763 (38%)		1.50	1.35, 1.68		0.32	0.11, 0.87	**0.029**
**Genus**			6,810			**<0.001**						6,290			**<0.001**			
*Acinetobacter*	470 (9.2%)	163 (9.7%)		*ref.*	—		*ref.*	—		374 (8.8%)	259 (13%)		*ref.*	—		*ref.*	—	
*Citrobacter*	370 (7.2%)	107 (6.4%)		0.83	0.63, 1.10		0.32	0.15, 0.67	**0.003**	333 (7.8%)	144 (7.1%)		0.62	0.48, 0.80		0.17	0.07, 0.43	**<0.001**
*Enterobacter*	356 (6.9%)	101 (6.0%)		0.82	0.61, 1.08		2.35	1.41, 3.91	**<0.001**	349 (8.2%)	108 (5.3%)		0.45	0.34, 0.58				
*Enterococcus*	428 (8.3%)	187 (11%)		1.26	0.98, 1.61		30.8	7.01, 155	**<0.001**	346 (8.1%)	267 (13%)		1.11	0.89, 1.40		0.87	0.46, 1.66	0.7
*Escherichia*	316 (6.2%)	37 (2.2%)		0.34	0.23, 0.49		0.26	0.11, 0.56	**<0.001**	313 (7.4%)	40 (2.0%)		0.18	0.13, 0.26		0.05	0.02, 0.14	**<0.001**
*Klebsiella*	578 (11%)	176 (10%)		0.88	0.69, 1.12		0.87	0.45, 1.60	0.7	591 (14%)	163 (8.0%)		0.40	0.31, 0.50		0.32	0.19, 0.56	**<0.001**
*Listeria*	207 (4.0%)	73 (4.4%)		1.02	0.74, 1.40		0.59	0.36, 0.95	**0.030**									
*Providencia*	92 (1.8%)	87 (5.2%)		2.73	1.94, 3.85		12.4	4.88, 32.2	**<0.001**	98 (2.3%)	81 (4.0%)		1.19	0.85, 1.67		1.35	0.84, 2.17	0.2
*Pseudomonas*	462 (9.0%)	111 (6.6%)		0.69	0.53, 0.91		1.78	1.06, 2.97	**0.029**	362 (8.5%)	209 (10%)		0.83	0.66, 1.05		0.74	0.50, 1.10	0.13
*Salmonella*	397 (7.7%)	84 (5.0%)		0.61	0.45, 0.82		0.02	0.00, 0.06	**<0.001**	341 (8.0%)	140 (6.9%)		0.59	0.46, 0.76				
*Serratia*	175 (3.4%)	61 (3.6%)		1.01	0.71, 1.41		0.26	0.10, 0.62	**0.003**	170 (4.0%)	66 (3.2%)		0.56	0.40, 0.77				
*Shigella*	277 (5.4%)	131 (7.8%)		1.36	1.04, 1.79		0.06	0.02, 0.21	**<0.001**	111 (2.6%)	61 (3.0%)		0.79	0.56, 1.12				
*Staphylococcus*	685 (13%)	195 (12%)		0.82	0.65, 1.04		1.85	0.71, 4.88	0.2	607 (14%)	273 (13%)		0.65	0.52, 0.80		1.05	0.56, 1.98	0.9
*Streptococcus*	320 (6.2%)	164 (9.8%)		1.48	1.14, 1.92					261 (6.1%)	223 (11%)		1.23	0.97, 1.57				
**WHO Risk**			6,810			**<0.001**						6,290			**<0.001**			
Critical	1,594 (31%)	385 (23%)		*ref.*	—					1,484 (35%)	495 (24%)		*ref.*	—				
High	1,208 (24%)	337 (20%)		1.16	0.98, 1.36					972 (23%)	569 (28%)		1.75	1.52, 2.03				
Medium	447 (8.7%)	195 (12%)		1.81	1.47, 2.21					351 (8.2%)	291 (14%)		2.49	2.06, 2.99				
NA	1,884 (37%)	760 (45%)		1.67	1.45, 1.92					1,449 (34%)	679 (33%)		1.40	1.23, 1.61				
**GLASS Pathogen**			6,810			**<0.001**						6,290			**<0.001**			
GLASS	4,009 (78%)	1,117 (67%)		*ref.*	—					3,439 (81%)	1,449 (71%)		*ref.*	—				
Non-GLASS	1,124 (22%)	560 (33%)		1.79	1.58, 2.02					817 (19%)	585 (29%)		1.70	1.50, 1.92				
**Clinical case**			6,154			**<0.001**						5,634			**<0.001**			
Community associated infections	1,670 (35%)	588 (41%)		*ref.*	—		*ref.*	—		1,184 (31%)	558 (31%)		*ref.*	—		*ref.*	—	
Healthcare associated infections	2,726 (58%)	817 (57%)		0.85	0.75, 0.96		0.19	0.09, 0.37	**<0.001**	2,360 (61%)	1,179 (66%)		1.06	0.94, 1.20		0.51	0.30, 0.86	**0.013**
Recurrent community infections	316 (6.7%)	37 (2.6%)		0.33	0.23, 0.47					313 (8.1%)	40 (2.3%)		0.27	0.19, 0.38				
**Human Specimen**			4,449			**<0.001**						3,933			**<0.001**			
CSF, sterile aspirates	828 (24%)	344 (33%)		*ref.*	—		*ref.*	—		555 (21%)	337 (27%)		—	—				
Sputum/tracheal	849 (25%)	181 (17%)		0.51	0.42, 0.63		0.14	0.06, 0.31	**<0.001**	725 (27%)	305 (25%)		0.69	0.57, 0.84				
Stool	538 (16%)	175 (17%)		0.78	0.63, 0.97					344 (13%)	133 (11%)		0.64	0.50, 0.81				
Urine	1,187 (35%)	347 (33%)		0.70	0.59, 0.84		0.08	0.03, 0.19	**<0.001**	1,081 (40%)	453 (37%)		0.69	0.58, 0.82				
**Animal Specimen**			3,636			0.24						3,398			0.086			
Milk	711 (26%)	236 (26%)		*ref.*	—					594 (27%)	353 (30%)		*ref.*	—		*ref.*	—	
Tissue	1,452 (53%)	455 (50%)		0.94	0.79, 1.13					1,116 (50%)	553 (47%)		0.83	0.71, 0.99		0.81	0.48, 1.36	0.4
Urine/Caecal	571 (21%)	211 (23%)		1.11	0.90, 1.38					520 (23%)	262 (22%)		0.85	0.69, 1.03		2.31	1.38, 3.92	**0.002**
**Environment Specimen**			3,715			0.30						3,431			**<0.001**			
Food	643 (23%)	178 (20%)		*ref.*	—					344 (15%)	195 (18%)		*ref.*	—				
Swabs	1,081 (38%)	343 (39%)		1.15	0.93, 1.41					943 (40%)	479 (45%)		0.90	0.73, 1.10				
Waste water	1,111 (39%)	359 (41%)		1.17	0.95, 1.43					1,083 (46%)	387 (36%)		0.63	0.51, 0.78				

^1^n (%).

^2^OR = Odds Ratio, CI = Confidence Interval.

Sector was significantly associated with identification performance (p = 0.010). In multivariable analysis, human health laboratories had significantly lower odds of acceptable identification results compared to animal health laboratories (aOR: 0.58, 95% CI: 0.46–0.71, p < 0.001). Environmental health laboratories (aOR: 1.10, 95% CI: 0.50–2.32, p = 0.8) and food safety laboratories (aOR: 0.51, 95% CI: 0.20–1.14, p = 0.13) did not differ significantly from animal health laboratories.

Participation in EQuAfrica cycles was strongly associated with identification performance (p < 0.001). Compared to laboratories participating in only one cycle, those participating in three cycles (aOR: 0.52, 95% CI: 0.29–0.92, p = 0.025) and five or more cycles (aOR: 0.22, 95% CI: 0.13–0.39, p < 0.001) had significantly lower odds of unacceptable results, indicating better performance. Participation in two cycles (aOR: 0.83, 95% CI: 0.47–1.46, p = 0.5) and four cycles (aOR: 0.76, 95% CI: 0.32–1.80, p = 0.5) were not significantly associated.

The use of CLSI standards (aOR: 0.84, 95% CI: 0.55–1.32, p = 0.4) or EUCAST standards (aOR: 1.54, 95% CI: 0.95–2.55, p = 0.084) was not significantly associated with identification performance compared to CA-SFM standards in multivariable analysis. However, utilization of automated methods was significantly associated with performance (p = 0.007). Laboratories not using automated methods had higher odds of unacceptable identification results compared to those using automated methods (aOR: 2.27, 95% CI: 1.27–4.19, p = 0.007), and laboratories with varying use had intermediate odds (aOR: 1.73, 95% CI: 1.03–3.04, p = 0.045).

In multivariable analysis, Gram-positive organisms were associated with significantly lower odds of unacceptable identification compared to Gram-negative organisms (aOR: 0.07, 95% CI: 0.02–0.22, p < 0.001). Significant genus-level associations were observed (p < 0.001), with *Escherichia* (aOR: 0.26, 95% CI: 0.11–0.56, p < 0.001), *Salmonella* (aOR: 0.02, 95% CI: 0.00–0.06, p < 0.001), *Shigella* (aOR: 0.06, 95% CI: 0.02–0.21, p < 0.001), and *Serratia* (aOR: 0.26, 95% CI: 0.10–0.62, p = 0.003) showing significantly lower odds of unacceptable results compared to *Acinetobacter*. Conversely, *Enterococcus* (aOR: 30.8, 95% CI: 7.01–155, p < 0.001), *Providencia* (aOR: 12.4, 95% CI: 4.88–32.2, p < 0.001), and *Enterobacter* (aOR: 2.35, 95% CI: 1.41–3.91, p < 0.001) had significantly higher odds of unacceptable results. *Listeria* (aOR: 0.59, 95% CI: 0.36–0.95, p = 0.030) and *Pseudomonas* (aOR: 1.78, 95% CI: 1.06–2.97, p = 0.029) also showed significant associations.

Clinical case type was significantly associated with identification performance (p < 0.001). Healthcare-associated infections had lower odds of unacceptable identification compared to community-associated infections (aOR: 0.19, 95% CI: 0.09–0.37, p < 0.001). Human specimen type was also significantly associated (p < 0.001), with sputum/tracheal aspirates (aOR: 0.14, 95% CI: 0.06–0.31, p < 0.001) and urine (aOR: 0.08, 95% CI: 0.03–0.19, p < 0.001) showing lower odds of unacceptable results compared to CSF/sterile aspirates.

#### AST performance.

For AST performance, several factors were significantly associated with acceptable results in the multivariate model (Table 5). Participation in more EQA cycles was associated with lower odds of acceptable AST performance, with laboratories participating in 3 cycles (aOR = 0.44, 95% CI: 0.19–0.98, p = 0.047), 4 cycles (aOR = 0.32, 95% CI: 0.11–0.92, p = 0.036), and 5 or more cycles (aOR = 0.17, 95% CI: 0.08–0.38, p < 0.001) showing significantly lower odds compared to those participating in only 1 cycle. Human Health Laboratories had lower odds of acceptable AST performance compared to Animal Health Laboratories (aOR = 0.55, 95% CI: 0.43–0.69, p < 0.001).

Automation methods were also associated with AST performance. Laboratories using non-automated methods had higher odds of acceptable AST performance compared to automated methods (aOR = 2.63, 95% CI: 1.26–5.75, p = 0.012), as did those with varying automation use (aOR = 2.29, 95% CI: 1.20–4.64, p = 0.016). Gram-positive organisms were associated with lower odds of acceptable AST performance compared to Gram-negative organisms (aOR = 0.32, 95% CI: 0.11–0.87, p = 0.029).

Significant variation in AST performance was observed across genera. Significant genus-level associations were observed (p < 0.001), with *Citrobacter* (aOR: 0.17, 95% CI: 0.07–0.43, p < 0.001), *Escherichia* (aOR: 0.05, 95% CI: 0.02–0.14, p < 0.001), and *Klebsiella* (aOR: 0.32, 95% CI: 0.19–0.56, p < 0.001) showing significantly lower odds of unacceptable results compared to *Acinetobacter*. *Enterobacter* and *Salmonella* did not remain in the final multivariable model. Clinical case type was significantly associated with AST performance (p = 0.013). Healthcare-associated infections had lower odds of unacceptable AST results compared to community-associated infections (aOR: 0.51, 95% CI: 0.30–0.86, p = 0.013). Among animal specimens, urine/caecal specimens had significantly higher odds of unacceptable AST results compared to milk (aOR: 2.31, 95% CI: 1.38–3.92, p = 0.002). Environment specimen type was significantly associated with AST performance (p < 0.001), with wastewater specimens showing lower odds of unacceptable results compared to food specimens (aOR: 0.63, 95% CI: 0.51–0.78, p < 0.001).

Both self-reported bacteriology capability and AST guideline used were not significantly associated with AST performance in the multivariate model, despite showing significance in univariate analyses.

## Discussion

Results from the EQuAfrica programme are important and effective contributions to the development and evaluation of the capacity of microbiology laboratories in antimicrobial resistance testing. The participating laboratories in this PT programme were from 14 low-income countries in Africa, according to the World Bank classification and as such were selected to receive UK Fleming Fund support. These laboratories faced many challenges, ranging from maintaining basic infrastructure such as uninterrupted power and water supplies, to limited funds for consistent supplies of basic essential laboratory consumables and communication services, such as internet connections. Only a few laboratories had automated instruments for bacterial ID and AST. The Fleming Fund programme supported laboratory quality management system strengthening to selected laboratories with a focus on enhancing bacterial ID and AST.

Antibiotic susceptibility testing results are critical for patient management and for public health purposes since data are routinely distributed to national departments of health and various surveillance networks such as WHO GLASS. The GLASS framework is responding to ongoing challenges in reporting of pathogen ID and AST; therefore, laboratories need to follow the recommendations in the Global Antimicrobial Resistance and Use Surveillance System set out in the GLASS-AMR Manual version 2.0 of 2021 [3]. The EQuAfrica programme, based on its EQA results, contributed to accurate and quality data submission for GLASS surveillance in these countries, and was supported by the Fleming Fund.

In our PT programme, there was no laboratory that scored zero per cycle; nevertheless, no improvement trend was documented over the eight cycles, and performances varied per sector and laboratory tier levels. Less than half the laboratories performed AST, but these did well with acceptable performance for Gram-negative organisms. We identified an improvement in pathogen ID and AST in the Cycle 4 which may be explained by the submission of repeat isolates in the panel used in this round, therefore participants had had prior exposure to similar organisms and resistance profiles. In consecutive cycles, different panel compositions of isolates and antimicrobial susceptibility testing challenges were submitted which may explain the decrease in performance in later EQA cycles. Non-adherence to guidelines was a major reason for poorly performing laboratories. All laboratories received technical and mentorship support on how to address non-conformances in each cycle which was reflected in an average score of 73% across all cycles.

The overall poor performance of testing for oxacillin in *S. aureus* isolates although better for cefoxitin, indicated a concern with laboratory methodology. Being able to detect methicillin-resistant *Staphylococcus aureus* (MRSA) in animal health and environmental samples is of public health importance and laboratories should be able to perform this test to the required standard [4,5]. These same laboratories were challenged later with a *Staphylococcus* isolate to assess whether laboratory performance had improved. In general, our data showed that laboratories enrolled in our programme showed improved performance over time although not to the expected level, thereby increasing the validity of the AMR data reported, especially by veterinary laboratories, unlike the report by Wong *et al.* for veterinary laboratories in some European countries that demonstrated problems with identifying AMR, indicating the need for continuous harmonisation of methodologies [5]. Similarly, the EQAsia programme established in 2020, at the same time as our programme, showed significant improvement of laboratory performance over time [16]. Some differences in performances were observed between human and animal sectors participants on this program [16].

In comparison with the WHO NICD PT programme for AMR provided to African national public health laboratories where AST results showed poor performance, our findings were more positive [7]. The poor performance in AST was, in part, due to the large number of misidentifications of pathogens leading to automatic zero scores for AST. This discrepancy suggests disparate intra-laboratory testing proficiency, potentially driven by variations in testing frequency, the complexity of interpretive criteria, or the systematic availability of quality control reference materials.

The WHO External Quality Assurance System Proficiency Testing Program for Antimicrobial Susceptibility Testing (EQAPT-AST) programme in 33 countries started in 1998 through March 2021 emphasised the importance of not only following susceptibility testing methods to show improved accuracy but also of assessing reporting accuracy [7]. In our programme, we only assessed performance in AST but not reporting accuracy which may be considered for the future improvement of the programme.

From a statistical perspective, a study by Morales *et al* recommended the use of contingency tables for measures of sensitivity, specificity and predictive values as useful in the analysis and interpretation of quality control results in clinical microbiology. This methodology could be useful in similar studies [8]. Improving laboratory reports to make them more valuable and reliable for clinicians will help to improve the health of the general population.

Implementation of a sustainable quality management system that qualifies for a recognised accreditation scheme would be the optimal solution and would provide confidence in the quality of laboratory results. None of the microbiology laboratories in our programme had been accredited by any of the recognised accreditation schemes, which may explain the lack of established laboratory quality management system. Laboratory assessments should emphasise the implementation of diagnostic stewardship programmes, which improve the utilisation of microbiological tests and results at clinical level.

The publication by Helwigh *et al* describes the results of the 2022 external quality assessment (EQA) of antimicrobial susceptibility testing (AST) by clinical laboratories that participated in the European Antimicrobial Resistance Surveillance Network (EARS-Net). Almost all laboratories achieved a good level of concordance with expected results [9]. For some pathogens, such as *P. aeruginosa, S. pneumoniae, E. coli* and *A. baumannii* there were lower levels of concordance in the interpretation of the antibiotics levofloxacin, benzylpenicillin, piperacillin-tazobactam and tobramycin, respectively [9]. Our study showed similar issues reflected in lower performances.

## Limitations

This programme covered a limited number of countries in Africa that received Fleming Fund support, however without adequate and comprehensive geographical coverage; selected laboratories demonstrated different capacity in performance of ID and AST. Countries that received Fleming Fund funding have not developed succession plan to continue with laboratory capacity development to support submission of accurate data on AMR surveillance.

## Conclusions

Our manuscript highlights that the use of EQA programmes in detecting gaps in laboratory processes. Improving and maintaining the quality and accuracy of pathogen ID and AST is vital for effective AMR surveillance and control. This report illustrates that poorly performed AST at the beginning of the programme improved over the course of the programme. It is critically important to build sustainable capacity for bacteriology testing, including AST, in national AMR reference laboratories and clinical surveillance sites across Africa. The implementation of a robust quality management system for AST will generate accurate AMR surveillance data and support the implementation of the GLASS system. This report emphasises the importance of continuing this PT programme for AMR in African countries for interpretation of AST for national, regional and global surveillance initiatives and overall public health.

## Supporting information

S1 Table Master antimicrobial susceptibility testing (AST) program mapping. (DOCX)
