# Supplementary material for: Laboratory performance of antimicrobial susceptibility testing for GLASS pathogens among participants in the EQuAfrica programme, 2021‒2025
Source: PLOS Glob Public Health. 2026 Oct 27;6(10):e0007478. doi: 10.1371/journal.pgph.0007478 (PMC13712707; doi:10.1371/journal.pgph.0007478)
Supplement: Supplementary file 1 [file pgph.0007478.s001.docx]

**Supporting file**

S1 Table. Master Antimicrobial Susceptibility Testing (AST) Program Mapping

| **Pathogen Name** | **Cycle / Round** | **Sector** | **Specimen Type / Scenario Description** |
| --- | --- | --- | --- |
| **Klebsiella pneumoniae** | Cycle 1 | Animal Health | Ear canal swabs: Isolated from a clinically ill dog previously diagnosed with otitis externa in Dakar, Senegal. |
| **Klebsiella pneumoniae** | Cycle 1 | Food & Env. | Food and Environmental laboratory sample. |
| **Klebsiella pneumoniae** | Cycle 1 | Human Health | Sputum: Isolated from a 62-year-old female with pneumonia associated with prolonged hospitalisation due to surgical complications. |
| **Enterococcus faecium** | Cycle 1 | Animal Health | Caecal content: Collected from a chicken at a poultry farm using avoparcin as growth promotion. |
| **Enterococcus faecium** | Cycle 1 | Food & Env. | Food and Environmental laboratory sample. |
| **Enterococcus faecium** | Cycle 1 | Human Health | Blood culture: Specimen collected from a bacteremic patient in the intensive care unit. |
| **Pseudomonas aeruginosa** | Cycle 1 | Animal Health | Uterine swab: Collected from a horse diagnosed with endometritis during routine biosecurity measures around mating. |
| **Pseudomonas aeruginosa** | Cycle 1 | Food & Env. | Food and Environmental laboratory sample. |
| **Pseudomonas aeruginosa** | Cycle 1 | Human Health | Wound swab: Deep wound swab collected from a patient in a long-term healthcare facility. |
| **Staphylococcus aureus** | Cycle 1 | Animal Health | Milk sample: Isolated during a routine visit to a cattle farm, identified as a case of bovine mastitis. |
| **Staphylococcus aureus** | Cycle 1 | Food & Env. | Food and Environmental laboratory sample. |
| **Staphylococcus aureus** | Cycle 1 | Human Health | Pus: Collected from a surgical wound showing pus oozing 2 weeks after hospital discharge. |
| **Escherichia coli** | Cycle 2 | Animal Health | Caecal content: Watery diarrhea and slight loss of condition observed among pig weaners. |
| **Escherichia coli** | Cycle 2 | Food & Env. | Food and Environmental laboratory sample. |
| **Escherichia coli** | Cycle 2 | Human Health | Mid-stream urine: Urine specimen collected from a 52-year-old female with a recurrent urinary tract infection. |
| **Acinetobacter baumannii** | Cycle 2 | Animal Health | Trans-tracheal wash: Collected from a 6-month-old colt examined for bronchopneumonia with lower airway bacteremic abnormalities. |
| **Acinetobacter baumannii** | Cycle 2 | Food & Env. | Food and Environmental laboratory sample. |
| **Acinetobacter baumannii** | Cycle 2 | Human Health | Blood for culture: Blood specimen from a patient in the intensive care unit with suspected bacteremia. |
| **Shigella flexneri** | Cycle 2 | Animal Health | Clam tissue: Clams collected from a wet market and investigated as a suspected source of human diarrhea. |
| **Shigella flexneri** | Cycle 2 | Food & Env. | Food and Environmental laboratory sample. |
| **Shigella flexneri** | Cycle 2 | Human Health | Stool: Patient presenting with watery diarrhea, vomiting, and mild dehydration. |
| **Enterococcus faecium** | Cycle 2 | Animal Health | Urine: Specimen collected from a dog with a recurrent urinary tract infection. |
| **Enterococcus faecium** | Cycle 2 | Food & Env. | Food and Environmental laboratory sample. |
| **Enterococcus faecium** | Cycle 2 | Human Health | Pericardial fluid: Collected from a pericardial effusion of a 79-year-old male who recently had a pacemaker inserted. |
| **Streptococcus agalactiae** | Cycle 3 | Animal Health | Tank milk sample: Food safety inspection of unpasteurised milk collected from five dairy farmers. |
| **Providencia stuartii** | Cycle 3 | Animal Health | Tissue from skin lesions: Dog presented with progressive anorexia, difficulty urinating, severe skin ulceration, and cellulitis following cystitis. |
| **Pseudomonas aeruginosa** | Cycle 3 | Animal Health | Ear swab: Dog admitted with head shaking, malodor, and ear discharge, previously treated unsuccessfully with aminoglycosides. |
| **Klebsiella aerogenes** | Cycle 3 | Animal Health | Well water sample: Fish farmer complained of fish skin infections, suspecting contaminated water as the source. |
| **Staphylococcus aureus** | Cycle 4 | Animal Health | Farm dust sample: Dust sample collected from a pig farm as part of a pilot AMR surveillance program. |
| **Staphylococcus aureus** | Cycle 4 | Food & Env. | Farm dust sample: Dust sample collected from a pig farm as part of a pilot AMR surveillance program. |
| **Staphylococcus aureus** | Cycle 4 | Human Health | Blood: Isolated from a 42-year-old man with septicemia following prolonged hospitalisation. |
| **Escherichia coli** | Cycle 4 | Animal Health | Tissue sample (pericardial sac): Acute fatal septicemia and severe pericarditis observed at a chicken farm. |
| **Escherichia coli** | Cycle 4 | Food & Env. | Tissue sample (pericardial sac): Acute fatal septicemia and severe pericarditis observed at a chicken farm. |
| **Escherichia coli** | Cycle 4 | Human Health | Midstream urine: Collected from a 47-year-old woman admitted to the urology ward with recurrent cystitis and urethral calculi. |
| **Klebsiella pneumoniae** | Cycle 4 | Animal Health | Composite sewage sample: Collected from a wastewater treatment plant receiving both farm runoffs and hospital waste. |
| **Klebsiella pneumoniae** | Cycle 4 | Food & Env. | Composite sewage sample: Collected from a wastewater treatment plant receiving both farm runoffs and hospital waste. |
| **Klebsiella pneumoniae** | Cycle 4 | Human Health | Urinary catheter segment: Isolated from a urinary catheter in a 58-year-old patient admitted to the burn intensive care unit. |
| **Acinetobacter baumannii** | Cycle 4 | Animal Health | Blood sample: Stray dog admitted with suspicion of rabies, presenting several wounds and a bloodstream infection. |
| **Acinetobacter baumannii** | Cycle 4 | Food & Env. | Environmental pathogen isolation. |
| **Acinetobacter baumannii** | Cycle 4 | Human Health | Tracheal aspirate: Collected from a 67-year-old diabetic patient hospitalised in the ICU for ten days following pneumonia complications. |
| **Salmonella isangi** | Round 2024-1 | Animal Health | Wall gecko droppings: Collected from a poultry farm as part of an environmental zoonosis surveillance investigation. |
| **Salmonella isangi** | Round 2024-1 | Human Health | Blood: Patient presented to the emergency department with abdominal cramps, diarrhea, fever, and chills. |
| **Enterococcus faecium** | Round 2024-1 | Animal Health | Poultry boot swab: Collected for monitoring during a zoonosis surveillance visit to a poultry farm. |
| **Enterococcus faecium** | Round 2024-1 | Human Health | Blood culture: Collected from a bacteremic patient in the oncology intensive care unit. |
| **Enterobacter cloacae** | Round 2024-1 | Animal Health | Wastewater sample: Collected from a field run-off stream as part of environmental surveillance according to UNEP guidelines. |
| **Enterobacter cloacae** | Round 2024-1 | Human Health | Broncho-alveolar lavage: Pathogen isolated from an intubated ICU patient with hospital-acquired pneumonia. |
| **no growth** | Round 2024-1 | Animal Health | Milk sample: Collected as part of a routine bovine herd mastitis investigation and assessed for contaminants. |
| **no growth** | Round 2024-1 | Human Health | Cerebrospinal fluid: Pathogen assessment for a 10-year-old boy presenting with headache, fever, vomiting, mild neck pain, and photophobia. |
| **Pseudomonas aeruginosa (MDRO)** | Round 2024-2 | Animal Health | Uterine swab: Mare diagnosed with endometritis during routine measures to ensure biosecurity around breeding. |
| **Pseudomonas aeruginosa (MDRO)** | Round 2024-2 | Food & Env. | White cheese samples: Pathogen isolated from homemade white cheese samples collected from an open-air market. |
| **Pseudomonas aeruginosa (MDRO)** | Round 2024-2 | Human Health | Wound swab: Deep wound swab collected from a patient in a long-term healthcare facility. |
| **Serratia marcescens** | Round 2024-2 | Animal Health | Screws: Post-surgery complications observed in a cat that underwent a tibial fracture repair. |
| **Serratia marcescens** | Round 2024-2 | Food & Env. | Composite sewage sample: Collected from a wastewater treatment plant where both farm run-offs and hospital waste accumulate. |
| **Serratia marcescens** | Round 2024-2 | Human Health | Intravenous catheter: Sample collected from an oncology patient with bacteremia in the intensive care unit. |
| **Klebsiella aerogenes** | Round 2024-2 | Animal Health | Ear swab: Dachshund diagnosed with canine atopic dermatitis with erythematous lesions and crusts on the ear pinna. |
| **Klebsiella aerogenes** | Round 2024-2 | Food & Env. | Well water sample: Investigated after a fish farmer complained of fish skin infections and suspected the water source. |
| **Klebsiella aerogenes** | Round 2024-2 | Human Health | Midstream urine: Patient presenting with urinary urgency, burning, and painful micturition. |
| **Staphylococcus aureus (D-test pos)** | Round 2024-2 | Animal Health | Trans-tracheal wash sample: Collected from a 6-year-old horse with lower airway abnormalities examined for bronchopneumonia. |
| **Staphylococcus aureus (D-test pos)** | Round 2024-2 | Food & Env. | Farm dust sample: Dust sample collected from a pig farm as part of a pilot AMR surveillance program. |
| **Staphylococcus aureus (D-test pos)** | Round 2024-2 | Human Health | Bronchial-alveolar lavage: Collected from a 32-year-old male presenting with fever and multiple pulmonary abscesses. |
| **Citrobacter freundii (ESBL)** | Round 2025-1 | Animal Health | Milk sample: A 3-year-old dairy cow showing fever, reduced appetite, and mastitis in one quarter of the udder. |
| **Citrobacter freundii (ESBL)** | Round 2025-1 | Food & Env. | Lettuce leaf: Pre-packaged ready-to-eat salads collected during routine food safety inspection. |
| **Citrobacter freundii (ESBL)** | Round 2025-1 | Human Health | Midstream urine: A 55-year-old female presenting with fever, dysuria, and lower abdominal pain. |
| **Staphylococcus saprophyticus** | Round 2025-1 | Animal Health | Wound swab: A 2-year-old neutered dog presenting with a swollen, erythematous paw and mild lameness from thorns. |
| **Staphylococcus saprophyticus** | Round 2025-1 | Food & Env. | Environmental swab: Swabs collected from a stainless-steel surface near a milk storage tank at a dairy facility. |
| **Staphylococcus saprophyticus** | Round 2025-1 | Human Health | Wound swab: A 2-year-old male presenting with localised redness, swelling, and purulent discharge from a cat bite. |
| **Streptococcus agalactiae** | Round 2025-1 | Animal Health | Milk sample: Dairy goat presenting with decreased milk production and swelling in the udder under poor hygiene conditions. |
| **Streptococcus agalactiae** | Round 2025-1 | Food & Env. | Milk sample: Suspected bacterial contamination identified during routine product testing at a dairy processing plant. |
| **Streptococcus agalactiae** | Round 2025-1 | Human Health | Knee joint aspiration: A 70-year-old male diabetic presenting with fever, chills, and left knee pain with a history of poor wound healing. |
| **Salmonella enteritidis** | Round 2025-1 | Animal Health | Stool: Puppy presenting with a history of lethargy, vomiting, and bloody diarrhea after eating raw chicken scraps. |
| **Salmonella enteritidis** | Round 2025-1 | Food & Env. | Swab: Traced to an inadequately sanitised equipment processing line following raw poultry contamination testing. |
| **Salmonella enteritidis** | Round 2025-1 | Human Health | Stool: A 28-year-old male presenting with fever, abdominal cramps, and watery diarrhea after eating undercooked chicken. |
| **Enterobacter cloacae complex** | Round 2025-2 | Animal Health | Wastewater sample: Field run-off stream where communal cattle graze, collected as part of environmental surveillance. |
| **Enterobacter cloacae complex** | Round 2025-2 | Food & Env. | Wastewater sample: Field run-off stream where communal cattle graze, collected as part of environmental surveillance. |
| **Enterococcus faecalis** | Round 2025-2 | Animal Health | Caecal content: Specimen collected from a poultry farm using avoparcin as growth promotion. |
| **Enterococcus faecalis** | Round 2025-2 | Food & Env. | Used poultry bedding/litter: Specimen collected from a poultry farm using avoparcin for growth promotion. |
| **Acinetobacter baumannii complex** | Round 2025-2 | Animal Health | Blood culture: Stray dog admitted with neurological symptoms, several wounds, and a bloodstream infection. |
| **Acinetobacter baumannii complex** | Round 2025-2 | Food & Env. | Factory surface swab: Dust sample collected from a meat processing factory to assess various pathogens. |
| **Listeria monocytogenes** | Round 2025-2 | Animal Health | Blood culture: Sheep displaying central nervous symptoms (circling, unilateral paralysis) that died of suspected encephalitis. |
| **Listeria monocytogenes** | Round 2025-2 | Food & Env. | Food samples: Food samples from an elementary school kitchen collected during an outbreak investigation. |
