# Supplementary material for: Laboratory performance of antimicrobial susceptibility testing for GLASS pathogens among participants in the EQuAfrica programme, 2021‒2025
Source: PLOS Glob Public Health. 2026 Oct 27;6(10):e0007478. doi: 10.1371/journal.pgph.0007478 (PMC13712707; doi:10.1371/journal.pgph.0007478)
Supplement: Supplementary file 2 [file pgph.0007478.s003.docx]

**Journal Requirements:**

1. Please amend your detailed Financial Disclosure statement. This is published with the article. It must therefore be completed in full sentences and contain the exact wording you wish to be published.

i. Please clarify all sources of financial support for your study. List the grants, grant numbers, and organizations that funded your study, including funding received from your institution. Please note that suppliers of material support, including research materials, should be recognized in the Acknowledgements section rather than in the Financial Disclosure.

EQuAfrica program was funded by the Fleming Fund and the UK Aid regional grant phase 1 through the principal recipient African Society for laboratory Medicine (ASLM). There is no grant number.

PO, FH and BvP were supported directly by ASLM

Agreement between ASLM and WITS HEALTH CONSORTIUM (WHC (Pty) for NICD was registered under TIN number 0030984717 (the "African Society for Laboratory Medicine") and supported OP, RB and MS involvements.

Agreement between Amref was executed simultaneously by supporting DM; IPD contract was performed and FP supported.

ii. State the initials, alongside each funding source, of each author to receive each grant. For example: "This work was supported by the National Institutes of Health (####### to AM; ###### to CJ) and the National Science Foundation (###### to AM)."

O Perovic, and R Badat received ASLM funding TIN 0030984717and supported by National Institute for Communicable Diseases (NICD); others:

iii. State what role the funders took in the study. If the funders had no role in your study, please state: “The funders had no role in study design, data collection and analysis, decision to publish, or preparation of the manuscript.”

The funder had no role in the study design

iv. If any authors received a salary from any of your funders, please state which authors and which funders.

All authors received funding from the Fleming Fund and UK Aid

2. For studies involving third-party data, we encourage authors to share any data specific to their analyses that they can legally distribute. PLOS recognizes, however, that authors may be using third-party data they do not have the rights to share. When third-party data cannot be publicly shared, authors must provide all information necessary for interested researchers to apply to gain access to the data. (https://journals.plos.org/plosone/s/data-availability#loc-acceptable-data-access-restrictions)

For any third-party data that the authors cannot legally distribute, they should include the following information in their Data Availability Statement upon submission:

1) A description of the data set and the third-party source

2) If applicable, verification of permission to use the data set

3) Confirmation of whether the authors received any special privileges in accessing the data that other researchers would not have

4) All necessary contact information others would need to apply to gain access to the data

All data is available as supplementary material. Information on facilities, participants and other disclosing data is anonymized.

3. Please ensure that your Ethics Statement is available in its entirety at the beginning of your Methods section, under a subheading 'Ethics Statement'.

As no human or animal material was used for this programme and the PT program followed the ISO 17043 standard, no ethical permission was required. Based on ISO 17043 standard requirements, individual participants’ data remained confidential and protected.

4. Please upload separate figure files in .tif or .eps format. Also, remove the figures from your manuscript file but keep the legends.

Figures are uploaded according to recommendation.

For more information about figure files please see our guidelines:

https://journals.plos.org/globalpublichealth/s/figures

https://journals.plos.org/globalpublichealth/s/figures#loc-file-requirements

If the reviewer comments include a recommendation to cite specific previously published works, please review and evaluate these publications to determine whether they are relevant and should be cited. There is no requirement to cite these works unless the editor has indicated otherwise.

Reviewers' comments:

Reviewer's Responses to Questions

Comments to the Author

1. Does this manuscript meet PLOS Global Public Health’s publication criteria? Is the manuscript technically sound, and do the data support the conclusions? The manuscript must describe methodologically and ethically rigorous research with conclusions that are appropriately drawn based on the data presented.

Reviewer #1: Yes

Reviewer #2: Yes

2. Has the statistical analysis been performed appropriately and rigorously?

Reviewer #1: Yes

Reviewer #2: Yes

3. Have the authors made all data underlying the findings in their manuscript fully available (please refer to the Data Availability Statement at the start of the manuscript PDF file)?

The PLOS Data policy requires authors to make all data underlying the findings described in their manuscript fully available without restriction, with rare exception. The data should be provided as part of the manuscript or its supporting information, or deposited to a public repository. For example, in addition to summary statistics, the data points behind means, medians and variance measures should be available. If there are restrictions on publicly sharing data—e.g. participant privacy or use of data from a third party—those must be specified.

Reviewer #1: Yes

Reviewer #2: Yes

4. Is the manuscript presented in an intelligible fashion and written in standard English?

PLOS Global Public Health does not copyedit accepted manuscripts, so the language in submitted articles must be clear, correct, and unambiguous. Any typographical or grammatical errors should be corrected at revision, so please note any specific errors here.

Reviewer #1: Yes

Reviewer #2: Yes

5. Review Comments to the Author

Please use the space provided to explain your answers to the questions above. You may also include additional comments for the author, including concerns about dual publication, research ethics, or publication ethics. (Please upload your review as an attachment if it exceeds 20,000 characters)

Reviewer #1: Dear Authors,

I had the privilege of being invited to review your manuscript on AST EQA served by the EQuAFRICA programme.

Congratulations on your comprehensive and excellent presentation of the results!

I have a few comments on your manuscript:

- please follow the European Organisation of External Quality Assurance Providers in Laboratory Medicine (EQALM) Guidelines for publishing about interlaboratory comparison studies (PubILC) to ensure that all relevant items are included in the manuscript (DOI: 10.1080/10408363.2024.2335202)

An overview of the European Organization for External Quality Assurance Providers in Laboratory Medicine (EQALM) is in line with our approach to delivering an EQA programme for bacteriology, reference No 6 is added.

- You do not mention whether the laboratories have received any support to help them improve their performance. It has previously been shown that EQA providers’ services have a greater influence on laboratory performance than national economic conditions. This should be clarified in the manuscript.

Yes, the Fleming Fund programme represents the UK Government’s investment into improving laboratory capacity for diagnosis and surveillance of antimicrobial resistance (AMR) in low-income countries through building laboratory capacity, collecting drug resistance data and sharing data to promote rational use of antimicrobials in human, animal and environmental fields. 14 countries received grants to support and improve their quality management systems (QMS) in addition to participating in the PT programme to measure performance in AMR testing. Added in the text 104-111

- Please reconsider the presentation of the figures, particularly the choice of colours for the bars and the labelling; some details in the figures are difficult to read. Best wishes!

This is addressed in the revised Figures.

Reviewer #2: Authors are appreciated for well-conducted research, clear presentation of results, and valuable findings with scientific and practical relevance.

However, a few points require further clarification to improve the overall understanding and interpretation of the study results and to strengthen the manuscript. I look forward to seeing this work published. I have some major and minor comments below:

Major comments:

1. How many laboratories showed continued improvement over the 8 EQA cycles? No figure or table shows the overall ID and AST testing results with improved results

There has been no significant improvement over this period rather than fluctuating between cycles for ID. Lines 285-286; 294-302 lines. These results highlight that performance was not uniform across cycles, with notable improvements in Cycle 4 and considerable drops in Cycle 5, possibly reflecting differences in pathogen complexity or laboratory familiarity with the tested organisms.

2. Figure 9 should be removed or corrected as needed; CLSI and EUCAST don’t recommend testing gentamicin against Pseudomonas aeruginosa and gentamicin against enterococcus species if it is high-level gentamicin resistance should be clearly explained for the readers' clarity

Figure is removed from the manuscript.

3. Any interventions or meetings/ workshops or seminars conducted by the technical advisory team to discuss and guide laboratories about incorrect results and educate laboratory staff? Should mention details
   a. . Post-cycle online workshops were organized and hosted by the regional EQA providers after each cycle to provide technical assistance for all participating laboratories. Strengths and challenges were addressed and provision of technical advice to address challenges was provided. As the part of the overall programme, mentors (subject matter experts) were also allocated by ASLM to provide on-site country support to laboratories and sentinel surveillance sites. Lines: 187-192
4. Methodology (Line 24) mentions “including all GLASS and other bacterial and fungal pathogens,” but none of the panels include any fungal pathogens?

We have included yeast in one survey but due to inconsistency of results between three providers we haven’t evaluated and weren’t included in the analysis. Fungi is removed from the list. Line 219 and Figure 1.

Minor Comments:

5. Proofreading required for lines 48-53 to improve clarity and correct spelling or clerical errors

Done

7. In Figure 4, were these laboratories scoring zero in EQA cycles the same throughout the study?

Figure 4 is removed. There were no laboratories that scored total zero in any of the cycle.

8. Environmental and food safety laboratories not routinely performing AST were assessed using the same PT panel? However, they may benefit from panels containing environmental or food-borne pathogens to better evaluate their competency.

Thank you for this comment. It is correct, we did not develop the programme to cater for the individual human, animal and environmental sectors, but rather we used the One Health approach where all sectors received the same samples. The project Technical Advisory Committee (TAC) was composed of experts in all sectors, and the organisms and AST requirements were adjusted based on sector. However, different case scenarios were developed for the same isolates when applied to the different sectors Table 2. Lines 135-136.

9. What could be the reasons for improved performance in cycle 4 but decreased performance in the last EQA cycle (line 186)?

Improvement was noticed as a reflection of support to laboratories from country Fleming Fund grants that enabled additional human resources, procurements and other issues. Explanation may be due to familiar composition of panels as repeats and inclusion of already announced GLASS organisms. Analysis is performed and resulted in Tables 3,4,5 and text: Lines 294-375.

10. Please clarify what “well performing” means on line 222 and specify the criteria used to identify these laboratories

Acceptable scores (3 and 4) were considered as well performing outcomes. We removed “well performing: statement.

11. Which tool was used to categorize labs in different categories into basic, intermediate, or advanced labs during initial assessment?

Participating laboratories were nominated by national authorities, prioritizing AMR reference laboratories and major surveillance sites in the human, animal and environmental health sectors to embrace a One Health approach. At the beginning of the enrollment, we sent a survey to laboratories to indicate capacity level and based on this self-assessment we categorized laboratories into tiers: advanced (comprehensive ID and AST, including resistance mechanism detection), intermediate (routine ID and AST), and basic (limited ID or AST). Table 3 and lines 246-248.

12. How were the samples sent to laboratories, in which form? Were they provided with a simulated patient’s history to guide them about suspected pathogens?

Freeze dried isolates distributed in each survey were accompanied by patient, animal and environmental case scenarios line 131.

6. PLOS authors have the option to publish the peer review history of their article (what does this mean?). If published, this will include your full peer review and any attached files.

Do you want your identity to be public for this peer review? If you choose “no”, your identity will remain anonymous, but your review may still be made public.

For information about this choice, including withdrawal consent, please see our Privacy Policy.

Reviewer #1: No

Reviewer #2: Yes: Noureen Saeed

[NOTE: If reviewer comments were submitted as an attachment file, they will be attached to this email and accessible via the submission site. Please log into your account, locate the manuscript record, and check for the action link "View Attachments". If this link does not appear, there are no attachment files.]

Figure Resubmissions:

Done

While revising your submission, we strongly recommend that you use PLOS’s NAAS tool (https://ngplosjournals.pagemajik.ai/artanalysis) to test your figure files. NAAS can convert your figure files to the TIFF file type and meet basic requirements (such as print size, resolution), or provide you with a report on issues that do not meet our requirements and that NAAS cannot fix.

After uploading your figures to PLOS’s NAAS tool - https://ngplosjournals.pagemajik.ai/artanalysis, NAAS will process the files provided and display the results in the "Uploaded Files" section of the page as the processing is complete. If the uploaded figures meet our requirements (or NAAS is able to fix the files to meet our requirements), the figure will be marked as "fixed" above. If NAAS is unable to fix the files, a red "failed" label will appear above. When NAAS has confirmed that the figure files meet our requirements, please download the file via the download option, and include these NAAS processed figure files when submitting your revised manuscript.
