# Supplementary material for: Laboratory performance of antimicrobial susceptibility testing for GLASS pathogens among participants in the EQuAfrica programme, 2021‒2025
Source: PLOS Glob Public Health. 2026 Oct 27;6(10):e0007478. doi: 10.1371/journal.pgph.0007478 (PMC13712707; doi:10.1371/journal.pgph.0007478)
Supplement: Peer review history

# Peer review: Decision Letter 0

**DOI:** 10.1371/journal.pgph.0007478.r001

**Authors:** Sadia Shakoor

14 May 2026

PGPH-D-26-01241

Laboratory performance of antimicrobial susceptibility testing for GLASS pathogens among participants in the EQuAfrica programme, 2021‒2025

PLOS Global Public Health

Dear Dr. Perovic,

Thank you for submitting your manuscript to PLOS Global Public Health. After careful consideration, we feel that it has merit but does not fully meet PLOS Global Public Health’s publication criteria as it currently stands. Therefore, we invite you to submit a revised version of the manuscript that addresses the points raised during the review process.

Please submit your revised manuscript by Jul 13 2026 11:59PM. If you will need more time than this to complete your revisions, please reply to this message or contact the journal office at globalpubhealth@plos.org. When you're ready to submit your revision, log on to https://www.editorialmanager.com/pgph/ and select the 'Submissions Needing Revision' folder to locate your manuscript file.

Please include the following items when submitting your revised manuscript:

- A letter that responds to each point raised by the editor and reviewer(s). You should upload this letter as a separate file labeled 'Response to Reviewers'.
- A marked-up copy of your manuscript that highlights changes made to the original version. You should upload this as a separate file labeled 'Revised Manuscript with Track Changes'.
- An unmarked version of your revised paper without tracked changes. You should upload this as a separate file labeled 'Manuscript'.

Guidelines for resubmitting your figure files are available below the reviewer comments at the end of this letter.

As the corresponding author, your ORCID iD is verified in the submission system and will appear in the published article. PLOS supports the use of ORCID, and we encourage all coauthors to register for an ORCID iD and use it as well. Please encourage your coauthors to verify their ORCID iD within the submission system before final acceptance, as unverified ORCID iDs will not appear in the published article. *Only*  the individual author can complete the verification step; PLOS staff *cannot*  verify ORCID iDs on behalf of authors.

We look forward to receiving your revised manuscript.

Kind regards,

Sadia Shakoor

Academic Editor

PLOS Global Public Health

**Journal Requirements:**

1. Please amend your detailed Financial Disclosure statement. This is published with the article. It must therefore be completed in full sentences and contain the exact wording you wish to be published.

i. Please clarify all sources of financial support for your study. List the grants, grant numbers, and organizations that funded your study, including funding received from your institution. Please note that suppliers of material support, including research materials, should be recognized in the Acknowledgements section rather than in the Financial Disclosure.

ii. State the initials, alongside each funding source, of each author to receive each grant. For example: "This work was supported by the National Institutes of Health (####### to AM; ###### to CJ) and the National Science Foundation (###### to AM)."

iii. State what role the funders took in the study. If the funders had no role in your study, please state: “The funders had no role in study design, data collection and analysis, decision to publish, or preparation of the manuscript.”

iv. If any authors received a salary from any of your funders, please state which authors and which funders.

2. For studies involving third-party data, we encourage authors to share any data specific to their analyses that they can legally distribute. PLOS recognizes, however, that authors may be using third-party data they do not have the rights to share. When third-party data cannot be publicly shared, authors must provide all information necessary for interested researchers to apply to gain access to the data. (https://journals.plos.org/plosone/s/data-availability#loc-acceptable-data-access-restrictions)

For any third-party data that the authors cannot legally distribute, they should include the following information in their Data Availability Statement upon submission:

1) A description of the data set and the third-party source

2) If applicable, verification of permission to use the data set

3) Confirmation of whether the authors received any special privileges in accessing the data that other researchers would not have

4) All necessary contact information others would need to apply to gain access to the data

3. Please ensure that your Ethics Statement is available in its entirety at the beginning of your Methods section, under a subheading 'Ethics Statement'.

4. Please upload separate figure files in .tif or .eps format. Also, remove the figures from your manuscript file but keep the legends.

For more information about figure files please see our guidelines:

https://journals.plos.org/globalpublichealth/s/figures

https://journals.plos.org/globalpublichealth/s/figures#loc-file-requirements

If the reviewer comments include a recommendation to cite specific previously published works, please review and evaluate these publications to determine whether they are relevant and should be cited. There is no requirement to cite these works unless the editor has indicated otherwise.

Reviewers' comments:

Reviewer's Responses to Questions

**Comments to the Author**

1. Does this manuscript meet PLOS Global Public Health’s publication criteria?

Reviewer #1: Yes

Reviewer #2: Yes

**********

2. Has the statistical analysis been performed appropriately and rigorously?

Reviewer #1: Yes

Reviewer #2: Yes

**********

3. Have the authors made all data underlying the findings in their manuscript fully available (please refer to the Data Availability Statement at the start of the manuscript PDF file)?

The PLOS Data policy

Reviewer #1: Yes

Reviewer #2: Yes

**********

4. Is the manuscript presented in an intelligible fashion and written in standard English?

Reviewer #1: Yes

Reviewer #2: Yes

**********

**Reviewer #1:**  Dear Authors,

I had the privilege of being invited to review your manuscript on AST EQA served by the EQuAFRICA programme.

Congratulations on your comprehensive and excellent presentation of the results!

I have a few comments on your manuscript:

- please follow the European Organisation of External Quality Assurance Providers in Laboratory Medicine (EQALM) Guidelines for publishing about interlaboratory comparison studies (PubILC) to ensure that all relevant items are included in the manuscript (DOI: 10.1080/10408363.2024.2335202)

- You do not mention whether the laboratories have received any support to help them improve their performance. It has previously been shown that EQA providers’ services have a greater influence on laboratory performance than national economic conditions. This should be clarified in the manuscript.

- Please reconsider the presentation of the figures, particularly the choice of colours for the bars and the labelling; some details in the figures are difficult to read.

Best wishes!

**Reviewer #2:**  Authors are appreciated for well-conducted research, clear presentation of results, and valuable findings with scientific and practical relevance.

However, a few points require further clarification to improve the overall understanding and interpretation of the study results and to strengthen the manuscript. I look forward to seeing this work published. I have some major and minor comments below:

Major comments:

1. How many laboratories showed continued improvement over the 8 EQA cycles? No figure or table shows the overall ID and AST testing results with improved results

2. Figure 9 should be removed or corrected as needed; CLSI and EUCAST don’t recommend testing gentamicin against Pseudomonas aeruginosa and gentamicin against enterococcus species if it is high-level gentamicin resistance should be clearly explained for the readers' clarity

3. Any interventions or meetings/ workshops or seminars conducted by the technical advisory team to discuss and guide laboratories about incorrect results and educate laboratory staff? Should mention details

4. Methodology (Line 24) mentions “including all GLASS and other bacterial and fungal pathogens,” but none of the panels include any fungal pathogens?

Minor Comments:

6. Proofreading required for lines 48-53 to improve clarity and correct spelling or clerical errors

7. In Figure 4, were these laboratories scoring zero in EQA cycles the same throughout the study?

8. Environmental and food safety laboratories not routinely performing AST were assessed using the same PT panel? However, they may benefit from panels containing environmental or food-borne pathogens to better evaluate their competency.

9. What could be the reasons for improved performance in cycle 4 but decreased performance in the last EQA cycle (line 186)?

10. Please clarify what “well performing” means on line 222 and specify the criteria used to identify these laboratories

11. Which tool was used to categorize labs in different categories into basic, intermediate, or advanced labs during initial assessment?

12. How were the samples sent to laboratories, in which form? Were they provided with a simulated patient’s history to guide them about suspected pathogens?

**********

what does this mean?). If published, this will include your full peer review and any attached files.

**Do you want your identity to be public for this peer review?**  If you choose “no”, your identity will remain anonymous but your review may still be made public.

For information about this choice, including consent withdrawal, please see our Privacy Policy

Reviewer #1: No

Reviewer #2: **Yes:**  Noureen Saeed

**********

[NOTE: If reviewer comments were submitted as an attachment file, they will be attached to this email and accessible via the submission site. Please log into your account, locate the manuscript record, and check for the action link "View Attachments". If this link does not appear, there are no attachment files.]

**Figure Resubmissions:**

# Author comment: Author response to Decision Letter 1

**DOI:** 10.1371/journal.pgph.0007478.r002

18 Sep 2026

Attachment Submitted filename: Response to Reviewers.docx

# Editor report: Decision Letter 1

**DOI:** 10.1371/journal.pgph.0007478.r003

**Authors:** Sadia Shakoor

30 Sep 2026

Laboratory performance of antimicrobial susceptibility testing for GLASS pathogens among participants in the EQuAfrica programme, 2021‒2025

PGPH-D-26-01241R1

Dear Prof Perovic,

We are pleased to inform you that your manuscript 'Laboratory performance of antimicrobial susceptibility testing for GLASS pathogens among participants in the EQuAfrica programme, 2021‒2025' has been provisionally accepted for publication in PLOS Global Public Health.

Before your manuscript can be formally accepted you will need to complete some formatting changes, which you will receive in a follow up email. A member of our team will be in touch with a set of requests.

Please note that your manuscript will not be scheduled for publication until you have made the required changes, so a swift response is appreciated.

IMPORTANT: The editorial review process is now complete. PLOS will only permit corrections to spelling, formatting or significant scientific errors from this point onwards. Requests for major changes, or any which affect the scientific understanding of your work, will cause delays to the publication date of your manuscript.

If your institution or institutions have a press office, please notify them about your upcoming paper to help maximize its impact. If they'll be preparing press materials, please inform our press team as soon as possible -- no later than 48 hours after receiving the formal acceptance. Your manuscript will remain under strict press embargo until 2 pm Eastern Time on the date of publication. For more information, please contact globalpubhealth@plos.org.

Thank you again for supporting Open Access publishing; we are looking forward to publishing your work in PLOS Global Public Health.

Best regards,

Sadia Shakoor

Academic Editor

PLOS Global Public Health

***********************************************************

Reviewer Comments (if any, and for reference):
